# Deep Learning for Transient Image Reconstruction from ToF Data

**DOI:** 10.3390/s21061962

**Published:** 2021-03-11

**Authors:** Enrico Buratto, Adriano Simonetto, Gianluca Agresti, Henrik Schäfer, Pietro Zanuttigh

**Affiliations:** 1 Department of Information Engineering, University of Padova, Via Gradenigo 6/b, 35131 Padova, Italy; enrico.buratto26@gmail.com (E.B.); adriano.simonetto@phd.unipd.it (A.S.); 2 R&D Center Europe Stuttgart Laboratory 1, Sony Europe B.V., Hedelfinger Str. 61, 70327 Stuttgart, Germany; Gianluca.Agresti@sony.com (G.A.); Henrik.Schaefer@sony.com (H.S.)

**Keywords:** Time-of-Flight, multi-path interference, depth estimation, transient imaging, denoising, deep learning

## Abstract

In this work, we propose a novel approach for correcting multi-path interference (MPI) in Time-of-Flight (ToF) cameras by estimating the direct and global components of the incoming light. MPI is an error source linked to the multiple reflections of light inside a scene; each sensor pixel receives information coming from different light paths which generally leads to an overestimation of the depth. We introduce a novel deep learning approach, which estimates the structure of the time-dependent scene impulse response and from it recovers a depth image with a reduced amount of MPI. The model consists of two main blocks: a predictive model that learns a compact encoded representation of the backscattering vector from the noisy input data and a fixed backscattering model which translates the encoded representation into the high dimensional light response. Experimental results on real data show the effectiveness of the proposed approach, which reaches state-of-the-art performances.

## 1. Introduction

In the last decades, there has been a surge of interest regarding range imaging technologies. These devices typically provide depth images, showing the distance of each scene point from the camera sensors. The applications of these systems and devices are widespread, as they can be used for augmented reality, face identification, gesture recognition [1], simultaneous localization and mapping [2], 3D modeling and reconstruction [3,4,5,6,7], autonomous driving [8,9] and even for navigation and landing on planetary bodies [10].

Some of the most common technologies comprehend stereo devices [11], that use the point of view of two different cameras to recover depth images, structured light scanners [12], that compute distance information relying on light patterns and LIDARs [13] and other Time-of-Flight (ToF) technologies [14], which use the light travel time for the recovery of depth information.

In this work, we will mostly focus on time-of-flight based devices—a Time-of-Flight (ToF) camera is a range imaging device that captures depth information in real time. The working principle of ToF cameras consists of illuminating the scene with a light source and then computing the time it takes for the light to travel from the source to the scene and back to the sensor. Recovering depth from time is then a straightforward operation. If the time is measured directly, for example, with a time to digital converter (TDC), we are working with direct ToF (dToF) cameras, while in the case the time is calculated from intensity measurements through correlation of illumination and sensor modulation, we are dealing with indirect ToF (iToF) cameras. We will focus on iToF cameras, which, compared to dToF, can estimate the depth with a smaller maximum range but with a higher lateral resolution. These aspects make them the better option for indoor acquisitions and the most adopted solution for ToF image sensors today. In the following section, we will give a mathematical introduction to the iToF principles.

### 1.1. iToF Cameras

The main idea behind the retrieval of depth information in ToF imaging is to use the fact that the speed of light is fixed. In direct ToF a very short light pulse is emitted towards the scene, is reflected and is finally gathered by a sensor; the depth is then inferred from the travel time. Indirect ToF makes use of the same idea, but with a different kind of illumination signal. It uses a periodic light modulation and retrieves the depth information from the phase displacement φ between the incoming light and an internal reference signal, according to the following relation:

(1) $$d=\frac{c\varphi }{4\pi f_{m}},$$

where $f_{m}$ is the modulation frequency and *c* is the speed of light.

In practice, the emitter sends a modulated signal i(t) with modulation frequency $f_{m}$ towards the scene. At the sensor’s side, the reflected light r(t) is correlated with the sensor sensitivity function s(t), that is, phase shifted by a factor θ for ToF measurements as explained below; the raw measurements $m_{\theta }$ of our camera are the result of these correlation operations. In the case of i(t) being a sine wave, we can express the reflected light as an attenuated and delayed version of the original signal $r\left(t\right)=\alpha i\left(t-\Delta t\right)=\alpha i\left(t-\frac{\varphi }{2\pi f_{m}}\right)$, where we expressed the delay Δt in terms of phase displacement. If we then consider a sensor sensitivity of the form $s\left(t\right)=1(\sin\left(2\pi f_{m}t\right)>0)$ and assume that the light bounces a single time inside the scene (quite a strong assumption as we will see) it holds that:

(2) $$m_{\theta }=\int_{0}^{T_{int}}r\left(t\right)s\left(t+\frac{\theta }{2\pi f_{m}}\right)dt,$$

with $T_{int}$ the integration time and θ the phase displacement applied to the sensor sensitivity.

By assuming $T_{int}\gg T_{m}=\frac{1}{f_{m}}$ (that is usually the case), we get the following closed-form solution of our integral [15]:

(3) $$m_{\theta }=I+A\cdot \cos\left(\varphi +\theta \right),$$

where *I* is the intensity of ToF signal, *A* is its amplitude and φ is the phase offset due to the scene depth by means of Equation (1).

From the raw measurement in (3), we want to recover *A*, *I* and φ. As shown in [15], it is sufficient to sample $m_{\theta }$ at 4 different known phase displacements θ of the sensor sensitivity $s(t+\frac{\theta }{2\pi f_{m}})$ in order to get the following relations:

(4) $$\begin{array}{cc} A & =\frac{1}{2}\sqrt{{\left(m_{0}-m_{\pi }\right)}^{2}+{\left(m_{\frac{3\pi }{2}}-m_{\frac{\pi }{2}}\right)}^{2}}, \end{array}$$

(5) $$\begin{array}{cc} \varphi & =\arctan2\left(\frac{m_{\frac{3\pi }{2}}-m_{\frac{\pi }{2}}}{m_{0}-m_{\pi }}\right), \end{array}$$

(6) $$\begin{array}{cc} I & =\frac{m_{0}+m_{\frac{\pi }{2}}+m_{\pi }+m_{\frac{3\pi }{2}}}{4}. \end{array}$$

As introduced by Gupta et al. [16], a quite convenient representation of the sinusoidal correlation function in (3) is the phasor notation. In practice, we can express the raw measurements in the following alternative way:

(7) $$v=Xe^{i\varphi }=Xe^{i2\pi f_{m}\Delta t}\in C,$$

where *X* corresponds to the amplitude and φ the phase of the original sinusoidal function.

We have considered the case in which the ToF signal is reflected only once inside the scene. However, in real scenarios it is highly likely for the light to be reflected multiple times, causing numerous light rays to arrive at the same pixel. This effect is called Multi-Path Interference (MPI). In this case, it is possible to generalize the above description by assuming that the resulting ToF signal is the summation of the different interfering signals, each one described as a phasor. Recall that sinusoidal signals with the same frequency, as well their phasor representation, are closed under summation [16]. As a consequence, a ToF measurement originated by MPI can be described as

(8) $$v=\int_{t_{min}}^{t_{max}}x\left(t\right)e^{i2\pi f_{m}t}dt,$$

where $t_{max}$ is the maximum time of flight of the considered interfering rays and x(t) is the so called backscattering distribution function, describing the strength of the interfering rays, given their times of flight. The MPI phenomenon described above is a non-zero mean error in ToF depth measurements, usually leading to an overestimation of depth. A key aspect of MPI distortion is its dependency on the geometry of the considered scene, which heavily influences the backscattering distribution function x(t). While having been widely studied [17,18,19,20], the problem is still challenging.

Other noise sources affecting iToF devices are photon shot noise, linked to the random nature of light, thermal noise due to sensor electronics and signal distortion issues, which are a consequence of non-idealities of the emitted light signals [12,21,22]. These errors, while still relevant, are easier to be tackled, and can be alleviated using calibration and filtering (e.g., bilateral filtering or total variation methods [23,24]).

As follows, we will introduce transient cameras, which are another range imaging technology strictly related to iToF; we will describe the relationship between the two and then continue by considering perks and limitations of transient sensors.

### 1.2. Transient Cameras

As previously remarked, the MPI effect is due to the multiple paths that single light rays follow after a reflection inside the scene. The integration done by an iToF sensor hides these multiple contributions and leads to an overestimation. As a matter of fact, if we were able to avoid such integration and instead capture the intensity of light arriving at the sensor at each time instant, we would be able to isolate the contributions due to different time of arrival of the incoming light reflections and therefore quickly understand what is the relevant information (the direct component) and what instead is noise (the global component).

**Figure 1:**
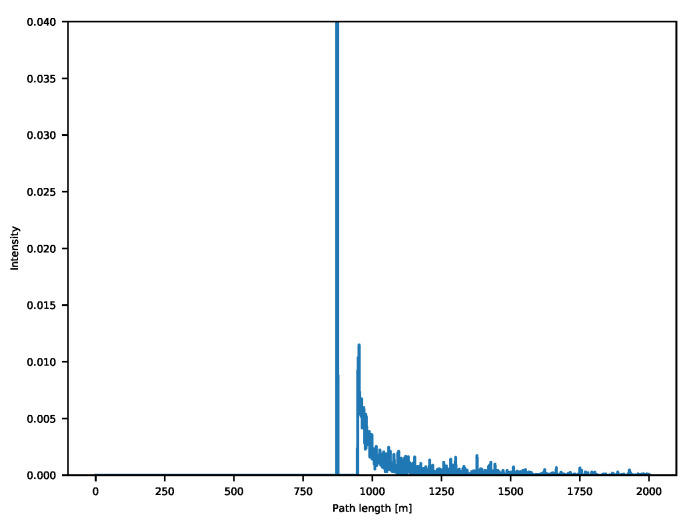
Example of a backscattering vector for a corner scene.

Transient cameras are relatively new devices that do exactly this. In practice, transient sensors are able to capture the incoming intensity of light at extremely high temporal resolutions. As we are working with the speed of light, current sensors need a temporal resolution in the order of the tens of picoseconds [25] for millimeter distance resolution. What we are getting in this way is the behavior of light through time. For each of the sensor pixels, we record the backscattering vector, which makes it easy to avoid any interference-related issue and provides additional insights regarding scene geometry, reflective properties of the captured objects and in theory could even allow to retrieve information of elements out of line of sight [26]. An example is shown in Figure 1.

The mentioned perks, while certainly appealing, come at a cost as transient cameras currently have a low spatial resolution and higher prices w.r.t. their ToF counterpart [25,27]. The research on transient cameras is quite active—in [28], the authors introduce a transient frequency transport equation, which helps simplifying transport analysis problems by tackling them in the frequency domain. More recently, in [29] a co-focal scanning procedure is used to tackle the task of non-line-of-sight imaging, while [30,31] tackle the task using the concepts of Fermat paths and of phasor fields, respectively. Nowadays however, transient cameras are mostly limited to research grade instrumentation, while ToF cameras are available as off-the-shelf products.

In this work, we propose to employ the underlying structure of transient images as a prior for MPI denoising. In practice, we propose to train a deep learning model for MPI correction, with a solution following a simplified backscattering model. To our knowledge, this is the first time that transient information is used for the task of MPI correction in a deep learning framework. Many authors in the literature proposed methods to correct MPI starting from the raw data acquired by a ToF camera or from noisy depth measurements [18,19,20,32]. The focus of this work is the development of a completely new approach for MPI correction based on the underlying structure of the backscattering vectors. The deep learning pipeline we propose is split into two main blocks: a predictive model, which learns the relation between the noisy iToF measurements and the encoded version of our transient data and a fixed model which translates the encoded information into the corresponding transient vector. While the network was developed under the strongly simplifying assumption that MPI is related to a backscattering vector composed by two peaks, one for the direct light reflection and a second peak summarizing the global light, the approach still reaches competitive performance with state-of-the-art approaches. The proposed pipeline is to our knowledge the first data-driven approach going in the direction of transient image reconstruction from noisy ToF data.

The remainder of this paper is articulated in the following way—in Section 2, we provide a review of the related literature. In Section 3, we describe the training pipeline and the idea behind our model in depth, while in Section 4 we show the datasets employed and then provide both qualitative and quantitative evaluation of our approach in Section 5. Finally, in Section 6 we draw our conclusions and mention some future developments.

## 2. Related Work

We will now describe some of the key works in the literature for MPI denoising, starting from approaches dealing with measurements at single or multiple frequencies, then focusing on some of the most recent deep learning methods which set the current state of the art and finally mentioning some other methods combining ToF with other 3D acquisition devices.

Many approaches rely only on standard measurements from ToF cameras. A single modulation frequency acquisition method is introduced by Fuchs et al. in [33], where a two bounces scenario on ideal lambertian surfaces is considered. In [34], they refine the approach by improving the reflection model and taking into account materials with multiple albedos. In [35], Jiménez et al. start from a similar setting and then use an iterative optimization algorithm to find the image which best fits the ToF measurements. The cited approaches show a nice performance but prove to be quite slow due to the high computation time of the algorithms employed, which makes them unfeasible for real-time applications.

The method in [36] by Freedman et al. is instead based on multi-frequency ToF acquisitions; they study MPI denoising as a LP minimization problem and propose an algorithm which works in real-time relying on a precomputed look-up table (LUT). The approach allows to estimate the backscattering information but the model is limited to a few peaks related to specular reflections. Bhandari et al. [37] introduce instead a closed-form solution for light rays bouncing a maximum of *K* times inside the scene, which however requires a high number of acquisitions at different frequencies (2K+1) to be implemented.

All the mentioned approaches restrict themselves to a simplified model, limiting the maximum amount of bounces, or requiring only specular reflections inside the scene. The need of more general models and the creation of bigger ToF datasets [19,38], opened the way for data-driven models, which set the current state-of-the-art. In [18] Son et al. designed a setup where a ToF camera is mounted on a robotic arm at acquisition time. They then employ two neural networks for the denoising, the first one *F* for mapping the ToF measurements to the correct range and the second *G* to correctly detect the object boundaries. Marco et al. [19] proposed an encoder-decoder convolutional neural network (CNN) architecture where the encoder was trained in an unsupervised way over real data, while the decoder instead was trained with supervision on a synthetic dataset they introduced. However, the approach is limited to single frequency data, thus limiting the MPI removal capabilities. In the following years, different architectures and techniques were investigated—in [24], the authors proposed a CNN for multi-frequency data with two separate branches: a coarse network analyzing the global structure of the scene at a low resolution and a fine one capturing the small details at a local level. In [38], the authors propose a two-stage architecture combining an encoder-decoder pipeline with a kernel prediction network. Up to that point however, the evaluation of the models was carried out mainly on synthetic data, with only [17,38] showing a qualitative evaluation on real images, and [24] showing the performance of their approach on a real dataset, but at the same time highlighting a clear gap between synthetic and real. Focusing on this issue, in 2019 Agresti et al. [20] provided two novel realistic ToF datasets and devised an Unsupervised Domain Adaptation (UDA) strategy based on adversarial learning which showed impressive performance both on synthetic and real world data. In 2020, Dong et al. [32] introduced a residual pyramid network, which focused on MPI patterns at different resolutions for a better prediction.

Table 1 shows a high-level of comparison between the main related works and the approach we introduce in this paper. To the best of our knowledge, this is the first work which exploits deep learning to reconstruct transient information starting from iToF data. The trend is to solve the MPI denoising task in the depth domain [20,24,32], with only SRA [36] trying to also predict the associated transient information. The proposed method is build on a sparse physical model for the MPI effect, similar to those adopted in [33,34,35,36,37], but employs a CNN for the predictions. It relies on iToF data acquired at multiple modulation frequencies by a standard ToF camera, such as many of the most recent best performing works in the field [17,20,24,36,38], and it turns out to be one of the simplest in terms of computational complexity.

On a different note, the works in [39,40,41] combine stereo and ToF measurements for MPI denoising. In [42,43,44], the authors propose a modified ToF light source which projects a set of different spatial patterns over the scene and use the additional spatial information to recover a clean ToF signal.

## 3. Proposed Approach

This section is devoted to an introduction and in depth description of the proposed deep learning approach for MPI denoising. Following other works in the literature [19,20,32], we decided to rely on deep learning models for this work as they are clearly outperforming other competing methods.

The main novelty we propose is the introduction of the transient information inside our training pipeline. Transient data as we mentioned in Section 1, is closely related to iToF information. Starting from Equation (8), and following the study done in [36], we will express the relation between raw iToF measurements and the corresponding backscattering into a simple matrix multiplication. Following that, we will introduce our model which takes in input raw iToF measurements in order to predict transient information.

### 3.1. The Transient Imaging Prior

Before proceeding with the model description, we will present a simplified representation for Equation (8) describing MPI in ToF acquisition. This simplified representation will be useful for the proposed method implementation. Equation (8) has an integral formulation, which, however, is not practical when trying to train a neural network. For this reason, we consider the discrete version of this equation by sampling the time interval of integration into *N* time steps. This allows to rewrite the equation as:

(9) $$v=\left[\begin{array}{ccc} e^{i2\pi f_{m}t_{0}} & \cdots & e^{i2\pi f_{m}t_{N-1}} \end{array}\right]\left[\begin{array}{c} x_{0}\\ \vdots \\ x_{N-1} \end{array}\right]=\Phi^{\prime }x,$$

where we isolated the scene impulse response inside the backscattering vector $x\in R^{N\times 1}$ and used the matrix $\Phi^{\prime }\in C^{1\times N}$ for the measurement model.

At the same time, it is quite useful to consider a set of acquisitions made at *M* different modulation frequencies, as the distortion pattern due to interfering rays is frequency dependent, it changes and therefore provides additional information regarding MPI distribution; at the same time, this can also help getting a longer unambiguous range while keeping the same accuracy in the depth domain [45].

A quite straightforward generalization of Equation (9) leads to the following expression:

(10) $$v=\left[\begin{array}{c} v_{0}\\ \vdots \\ v_{M-1} \end{array}\right]=\left[\begin{array}{ccc} e^{i2\pi f_{0}t_{0}} & \cdots & e^{i2\pi f_{0}t_{N-1}}\\ \vdots & \ddots & \vdots \\ e^{i2\pi f_{M-1}t_{0}} & \cdots & e^{i2\pi f_{M-1}t_{N-1}} \end{array}\right]\left[\begin{array}{c} x_{0}\\ \vdots \\ x_{N-1} \end{array}\right]=\Phi x,$$

where $v\in C^{M\times 1}$ is the stack of the raw camera measurements in the complex domain at different modulation frequencies, while $\Phi \in C^{M\times N}$.

In conclusion, the problem we are dealing with is the following: given the raw measurements v and matrix Φ, we want to recover the backscattering vector x. The hereby presented problem is heavily under-constrained as N≫M, and has an infinite amount of solutions.

### 3.2. Training Pipeline

**Figure 2:**
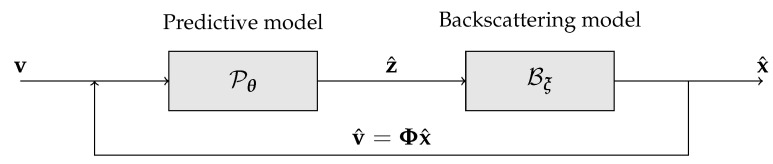
Structure of the proposed approach.

Deep neural networks can have issues when handling high-dimensional data [46,47], and this is exactly the case of transient information; a backscattering vector can easily have a few thousand entries in the temporal direction against a handful of iToF measurements, a problem that can make the training of the architecture hard if not impossible. In order to solve this issue, we decided to split the backscattering estimation task in two parts as shown in Figure 2. On one side we have the backscattering model, which takes care of the dimensionality reduction, while on the other we have the predictive model, the true deep learning backbone of the approach. The learnable predictive model maps the iToF measurements into a low dimensional space that is then expanded into the transient information by the Backscattering model. This allows to greatly reduce the dimensionality of the deep network output space making the training of the model feasible.

As follows, we will describe in depth the two components and finally conclude the section considering the loss functions used for training.

#### 3.2.1. Backscattering Model

The main task of the backscattering model is to compact the high dimensional transient information into a representation that is easier to handle. Basically, the task of this module $B_{\xi }$ is to map a latent variable z into the respective backscattering vector x:

(11) $$\begin{array}{cc} B_{\xi }:\;R^{L} & \to D_{x}\subseteq R^{N}, \end{array}$$

(12) $$\begin{array}{cc} z & \to x=B_{\xi }\left(z\right), \end{array}$$

where L≪N, ξ are in principle some trainable parameters and $D_{x}$ is the domain of all possible backscattering vectors. In more general settings, $B_{\xi }$ could be a generative model such as a generative adversarial network (GAN) or a Variational Autoencoder offering a precise mapping between a low-dimensional domain and the transient data. In practice however, since our task is MPI denoising, we really just need an accurate localization of the direct component (the first peak), while for the second global component a more concise encoding can suffice.

For our implementation, we decided to use a simple model for the backscattering vector, where just the two-rays interfering case is taken in consideration. This choice is motivated by the practical consideration that in real scenarios the first and second order reflections are the ones containing the largest part of the energy of the backscattering vector [48]. For this reason, we used as backscattering model a deterministic mapping from a 4-dimensional z vector ($z\in R^{4}$) to an approximated version of the backscattering information. More in detail, the 4 values of the z representation are the amplitudes and the path lengths of the first and the second interfering rays. The backscattering model has the task of converting these 4 values to the approximated backscattering vector that will be equal to zero on each entry apart from two peaks related to the first and the second interfering rays.

#### 3.2.2. Predictive Model

The deep learning part in our pipeline is the predictive model. Given an input matrix of raw iToF measurements at different modulation frequencies, it outputs the corresponding values in the latent domain Z. In practice, the predictive model is a highly non-linear function $P_{\theta }\left(\cdot \right)$, with parameters θ, that takes in input the vector v and produces an estimation of the corresponding vector z, that we will call $\hat{z}$.

**Figure 3:**
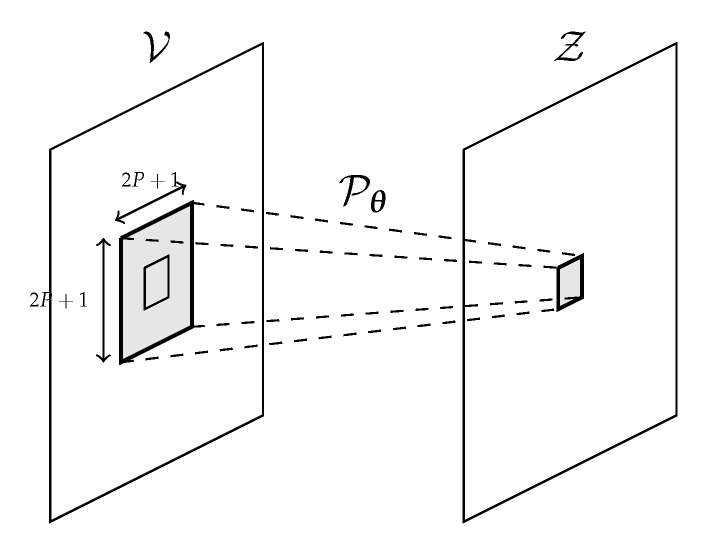
Predictive model working at a local level.

In order to better exploit the spatial information for the prediction on each pixel, we consider a local neighbourhood around the pixel itself of size (2P+1)×(2P+1) as in Figure 3, where *P* is a parameter that we experimentally set to 1 (each window is therefore 3×3).

**Figure 4:**
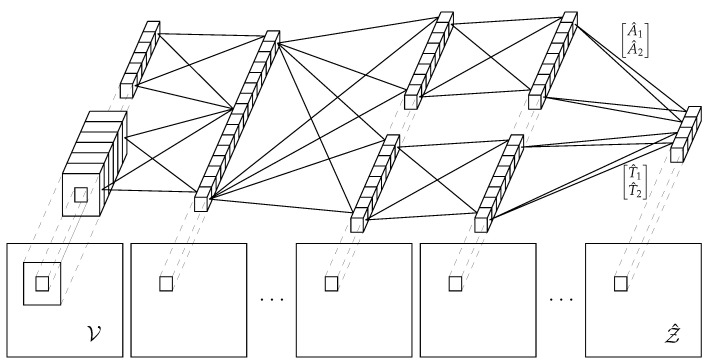
Predictive model structure.

In Figure 4, we show the shape of the proposed network. We employ a Convolutional Neural Network whose first layer combines a weight kernel which retrieves information from each pixel, and another small (2P+1)×(2P+1) kernel centered around the pixel itself which takes care of the local information. The rationale behind this model is that even though local spatial information is quite important, at the same time it is crucial to give a great importance to the data carried by the central pixel itself. As we will show in Section 5, the spatial information ensures a slightly better performance, but the central pixel itself conveys already a degree of information which is sufficient for an accurate prediction.

We want to bring to the attention of the reader that the strength of the approach does not rely for the most part on the quite reduced local information we provide (the kernels we employed are only 3×3) but on the global information that is inherently carried by each pixel. Other works in the literature relied on quite complex training structures in order to consider this critical piece of information as for example in [24], where a coarse network was proposed that considers the global structure of the scene, or in [32], where a pyramid structure tries to predict MPI at different resolution levels. The approach we propose does not need any complex addition, since the global information is already present in the form of transient data and already conveys the information regarding the scene structure. Even if the two-peaks representation we consider may seem quite rough, it is still enough for an accurate depth estimation as we will prove in Section 5.

#### 3.2.3. Training of the Deep Learning Model

The training has been performed using a combination of two losses: a standard supervised loss and a soft constraint which made sure the predictions were consistent with the model defined in Equation (10). The latter, called measurement loss, ensures that our prediction makes sense according to the raw iToF measurement we gave in input. Since the matrix Φ is known, for any predicted backscattering vector $\hat{x}$, we can compute the corresponding vector $\hat{v}$, which must be equal to the one we had in input for the prediction to make any sense. In other words we can write:

(13) $$L_{m}\left(v,\Phi \hat{x}\right)=∥v-\Phi \hat{x}∥=∥v-\hat{v}∥.$$

This loss, of course, only ensures a soft constraint since as mentioned the problem of Equation (10) is quite ill-conditioned. In order to get a meaningful result we therefore make use of a reconstruction loss, which simply ensures that our prediction $\hat{x}$ matches with the ground truth x. While being a simple supervised loss, it still presents a non trivial challenge as it is not that straightforward to define a suitable distance measure between sparse, high-dimensional vectors. Some common choices like the MSE or MAE quickly fail as we only have two meaningful values along a plateau of null entries. We need to define a loss function which amplifies the error in the case predicted and true peaks have different time and intensity components, and that at the same time keeps in lower consideration the null entries.

The main idea is to treat the two backscattering vectors as two different Probability Mass Functions (PMFs) and measure their statistical distance. The two distributions are defined as follows:

(14) $$p_{x}\left(n\right)=\frac{x_{n}}{X_{sum}}\;p_{\hat{x}}\left(n\right)=\frac{{\hat{x}}_{n}}{X_{sum}}\;\mathrm{where}\;X_{sum}=\sum_{n=0}^{N-1}x_{n},$$

where we normalize for the ground truth values in order to avoid divisions by zero issues when we have all-zero predicted vectors. The distance between the two is then computed using a modified version of the Earth Mover Distance (EMD) which, unlike some other divergence measures such as the Kullback-Leibler or Jensen-Shannon ones, does not require the two PMFs to have a common support. The standard EMD is defined as follows:

(15) $$EMD\left(p_{x},p_{\hat{x}}\right)=\sum_{n=0}^{N-1}\left|P_{x}\left(n\right)-P_{\hat{x}}\left(n\right)\right|,$$

where $P_{x}\left(n\right)$ and $P_{\hat{x}}\left(n\right)$ are the cumulative mass functions of the original distributions. Starting from the previous expression, we define the reconstruction loss between original and predicted backscattering vector according to a weighted Earth Mover Distance ($EMD_{w}$) as below:

(16) $$L_{r}\left(x,\hat{x}\right)=\frac{1}{NX_{sum}}EMD_{w}\left(p_{x},p_{\hat{x}}\right)=\frac{1}{NX_{sum}}\sum_{n=0}^{N-1}w_{n}\left|c_{n}-{\hat{c}}_{n}\right|,$$

with $c_{n}$ and ${\hat{c}}_{n}$ the cumulative functions of our backscattering vectors:

(17) $$c_{n}=\sum_{n^{\prime }=0}^{n}x_{n^{\prime }}\;{\hat{c}}_{n}=\sum_{n^{\prime }=0}^{n}{\hat{x}}_{n^{\prime }},$$

while the weights $w_{n}$ are computed as:

(18) $$w_{n}=\frac{1}{W}\sum_{k=0}^{W-1}\left|c_{n-k}-{\hat{c}}_{n-k}\right|,$$

with *W* a suitably sized window that, in our experiments, was set to 100. The reason for this modification was due to the fact that it is quite hard for the network to distinguish between direct and global components when the two peaks are very close one to the other; what tends to happen is that the first peak obscures the other, leading to predicting a single peak. The solution proposed in Equation (18) consists in giving more importance to elements which are preceded by other non-zero samples, thus balancing out the importance given to direct and global components.

#### 3.2.4. Bilateral Filtering

To further improve performances, an additional step was included in the pipeline in order to deal with zero-mean error sources. In practice, the predicted depth goes through a bilateral filter to get the final prediction. The parameters of the filter were experimentally set to $\sigma_{d}=0.05$ and $\sigma_{s}=10$, where the first value corresponds to the standard deviation of the kernel in the depth domain, and the second to the spatial one instead.

## 4. Training and Test Datasets

For the supervised optimization of the proposed approach we need a training set containing raw ToF data together with the corresponding ground truth transient data. Note that from geometrical considerations it is clear that the true depth value is always associated to the shortest returned path that corresponds to the direct component, and therefore depth information can be easily extracted from the transient scene. The acquisition of a real dataset with transient ground truth, however, is quite a complex and time consuming task; since no publicly available datasets of the kind exist, we had to rely on synthetic data.

For the training of the approach, we relied on the FLAT synthetic dataset introduced in [38], which contains transient data. At first, we applied a depth equalization procedure, in order to obtain a final distribution that is as uniform as possible. Then, the data was processed as discussed in Section 3.2.1 with the addition of a clipping operation for the intensity of the second peak, whose maximum value could be $h_{2}\leq 0.8h_{1}$, with $h_{1}$ and $h_{2}$ the intensity of the two peaks. Finally, the input iToF values were computed from the compressed transient information using the measurement model described in Equation (10) with modulation frequencies of 20,50 and 60 MHz. After the processing the data was then split into a training and a validation set, made of 211200 and 2064 3×3 patches respectively. Note that no test set was built at this phase since the testing will be performed on real images.

The performance in the depth estimation provided by the proposed approach is evaluated on real-world scenes where no transient data is given. Since our objective is MPI denoising, transient data is not required at the testing phase, all that is needed are the raw measurements at the desired modulation frequencies and the corresponding ground truth depth maps. From the predictions on the selected dataset, we can focus on the first peak and use that to estimate the depth value of each pixel. The real-world datasets on which we carry out our analysis are the three real ToF datasets $S_{3},S_{4},S_{5}$ provided by Agresti et al. in the works [20,24]. All three datasets have been captured in a laboratory environment without external illumination using the SoftKinetic ToF camera DS541 at multiple modulation frequencies. For each scene, they provide unwrapped phase, amplitude and intensity, as well as depth ground truth. The datasets are in the depth range between 58 and 203 cm.

In Table 2 we can see the resolution, number of images and acquired modulation frequencies of the three datasets.

In particular, the dataset $S_{3}$ will be used for validation while $S_{4}$ and $S_{5}$ will be our test sets. Notice that, for all three considered datasets, we used only the data acquired at 20, 50 and 60 MHz as input for the proposed method.

## 5. Experimental Results

In this Section we are going to present some experimental results obtained running the proposed approach for backscattering vector estimation.

The proposed network was trained on the simulated dataset described in Section 4 using the Adam optimization algorithm. The entire dataset was divided into batches of 1024 samples each, and the gradient at each iteration was computed on a single batch. We run the training for a total number of E=2000 epochs on a Nvidia GeForce GTX 1060 GPU. The overall time required was around 6 h. To account for the noise always present in any real-world ToF measurements, at each iteration a gaussian zero-mean random noise is added to the real and imaginary parts of the simulated raw ToF data:

(19) $$v=\Phi \;x+\eta \;\;\eta ∼N(0,\sigma_{v}^{2}).$$

The Gaussian noise is independent and identically distributed across all the samples and across all the acquired phasors at the different modulation frequencies. It is also independently added to the real and complex component. Changing the noise at each iteration helps avoiding overfitting and acts as a form of regularization. The network never sees the same exact input data more than once. Moreover, it helps the network in learning to denoise the input data, giving more importance to the more stable relationships between the acquired phasors and less to small fluctuations around the average. In Section 5.1 we will show this in more detail; for all other experiments, unless otherwise stated, we will use noise with a standard deviation $\sigma_{v}=0.02$. The choice of this value was performed as mentioned in Section 5.1.

The best set of weights are chosen according to the network performance on the real dataset $S_{3}$, which we employ as a realistic validation set. The testing is then carried out from a qualitative and quantitative point of view on the two real datasets $S_{4}$ and $S_{5}$ provided by Agresti et al. [20,24]. As anticipated in Section 4, the evaluations focus on the degree of MPI correction, while a few comparisons concerning the reconstruction of the transient component are reported in Section 5.1. The metric used to quantify the error in the depth domain is the Mean Absolute Error (MAE).

**Figure 5:**
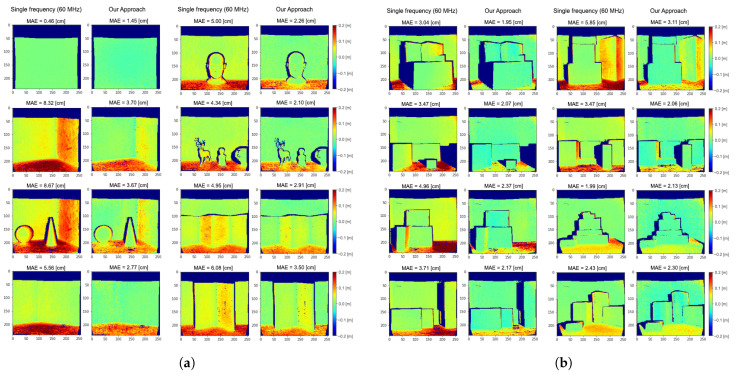
Depth error maps on the real datasets $S_{4}$ (**a**) and $S_{5}$ (**b**) obtained applying our method and a single frequency prediction at 60 MHz. Blue colour indicates depth underestimation, while red colour indicates depth overestimation. The dark blue areas are those for which we do not have ground truth depth available. The Mean Absolute Error (MAE) for each scene is also reported.

Figure 5 reports the depth error maps obtained applying our method on the real datasets $S_{4}$ and $S_{5}$. The average MAEs for raw ToF measurements at 60 MHz are respectively 5.43 and 3.62cm for $S_{4}$ and $S_{5}$; these errors are reduced to 2.60 and 2.12cm for the two datasets by the proposed method.

**Figure 6:**
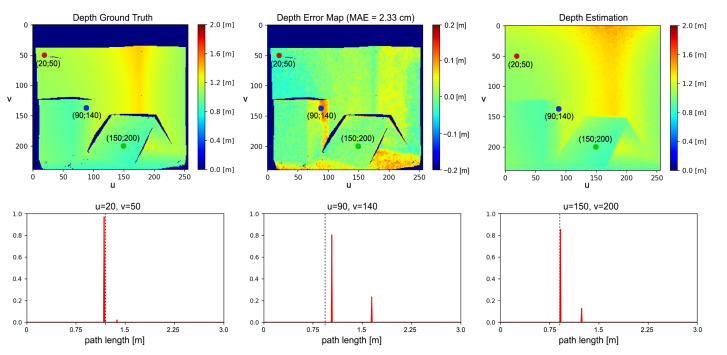
Network prediction for selected pixels in an image. The dashed lines correspond to the depth ground truth values while the red plots indicate the predicted backscattering vectors.

Even if we are considering only two reflections, experimental results show good MPI compensation capabilities, confirming that many real-world cases can be well approximated by a two components reflection model. This is due to the fact that, since the light power decays with the square of the distance, higher order reflections reaching the camera are very dim. Moreover, real lambertian surfaces present always a fraction of specular reflections and thus our assumption in first approximation holds also for those surfaces. From Figure 5 it is possible to see how our approach removes most of the MPI on wall surfaces and reduces it in proximity of edges. The large amount of MPI on the floor surfaces is also consistently reduced even if some depth reconstruction errors are still present. These areas are probably subject to more complex reflection patterns and are therefore more error prone. Looking at the network output at some significant points (Figure 6), it is evident that it has learnt to discriminate between MPI-free and MPI-affected pixels, introducting the global component only when it is necessary to compensate for the MPI effect and providing a more reliable depth estimation.

**Figure 7:**
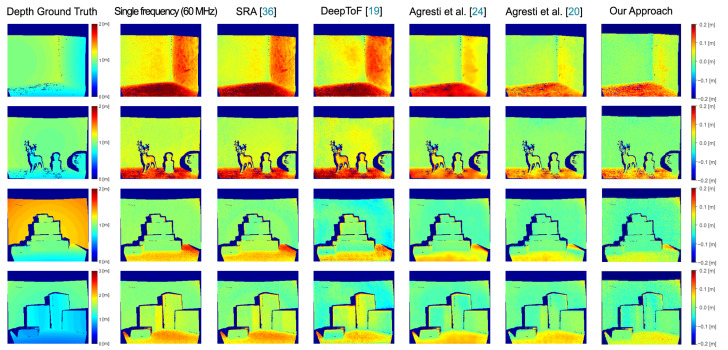
Qualitative comparison between several state-of-the-art MPI correction algorithms on some real scenes sampled from $S_{4}$ and $S_{5}$. On the left side the depth ground truth is shown, while the others display the error between the prediction of each method and the ground truth.

We have performed a thorough consistent comparison between different state-of-the-art algorithms for MPI compensation, studying how our approach behaves with respect to the others techniques. The compared algorithms are the SRA method proposed by Freedman et al. [36], DeepToF proposed by Marco et al. [19] and the two methods proposed by Agresti et al. [20,24]. Table 3 reports the MAEs and the relative errors for the compared algorithms on the real datasets $S_{4}$ and $S_{5}$, while Figure 7 shows a qualitative comparison between the competing approaches. The relative errors are computed as the ratio between the MAE of the approach and that of the reconstruction based on the highest input frequency (60 MHz for all the approaches but for DeepToF [19], which uses 20 MHz). From Figure 7 it is possible to notice how our approach achieves a much better MPI reduction on the floor and wall surfaces if compared with SRA and DeepToF. It also outperforms [24] and achieves results very similar to [20] even if no real depth acquisitions have been used for the training.

**Figure 8:**
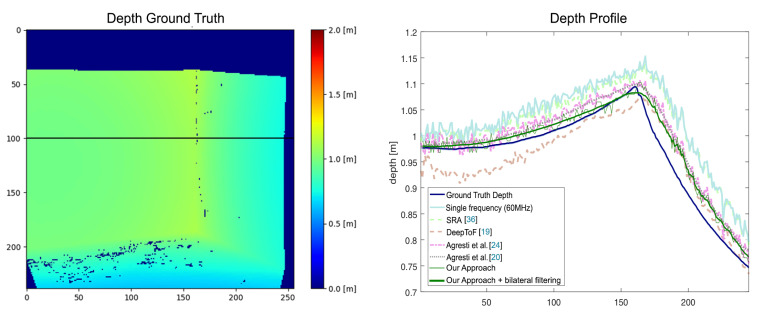
Depth profile estimation in proximity of a corner. The left plot reports the depth ground map, while the right one compares with our approach the depth profile over the highlighted line on the left image, estimated by different state-of-the-art MPI correction algorithms.

On real-world scenes our approach achieves performance comparable to the other state-of-the-art algorithms. It produces an error of 2.60cm on $S_{4}$ and an error of 2.12cm on $S_{5}$, performing better than all other methods, with the exception of the unsupervised domain adaptation technique of Agresti et al. [20] which produces test errors of respectively 2.36 and 1.66cm. We stress the fact that the unsupervised domain adaptation technique has been adapted using unsupervised real data similar to the one in $S_{4}$ and $S_{5}$, while our approach relies only on a completely different synthetic training dataset. It is remarkable to notice that our method clearly outperforms the SRA method [36], which is the one adopting the most similar setup to ours. We both acquire data at three modulation frequencies and use a physical model to describe the MPI effect under the specular reflections assumption. The MAEs on datasets $S_{4}$ and $S_{5}$ obtained by SRA are respectively 5.11 and 3.37cm. Figure 8 shows the depth profiles estimated in proximity of a corner using the compared algorithms. Also in this case our network is able to reconstruct depth values which closely resemble the ground truth.

Concerning the complexity of the compared algorithms, note that our approach is able to achieve state-of-the-art performance using around 22k learnable parameters (14k in the version without the exploitation of the spatial correlation), approximately an order of magnitude less with respect to the 145k parameters required by the two works of Agresti et al. [20,24]. The DeepToF method [19] requires even more parameters (330k). For SRA [36] the complexity is estimated by the size of the associated look-up table (LUT). Fixed the number *L* of discretization steps for the input raw iToF measurements, the size of the LUT grows as $L^{4}$.

As a final study, we decided to investigate the goodness of the prediction of our network for the intensity and time components of the two predicted peaks (corresponding to direct and global component). Since the $S_{3},S_{4}$ and $S_{5}$ dataset do not have transient information, we relied on the synthetic validation dataset we introduced in Section 4 in order to compare our approach to [36], which is the only one among the compared approaches that estimates the second peak. Comparing the accuracy of the reconstruction of the global component is quite straightforward—from Table 3 we can retrieve the depth information (which is linked to the time displacement) and we can see that our method reaches significantly better results (2.60 and 2.12cm on the $S_{4}$ and $S_{5}$ datasets respectively vs. 5.11 and 3.37 of [36]), while concerning the intensity values of the first peak we obtained a MAE of 0.0783 for our method against 0.1905 for SRA. For the second peak, since it is not always present, we considered the capability of the approaches to correctly detect its presence with the well known precision-recall measures. The precision (number of pixels correctly identified as having a second peak over the total amount that have it), is 0.945 for our approach against 1 for SRA. However, these results are due to the fact that SRA overestimates the presence of the second peak as shown by the recall measure (number of pixels correctly identified as having a second peak divided by the total amount of peaks identified), with a result of 0.839 for our approach against 0.675 for SRA. Our approach also better estimates the intensity of the second peak with a MAE of 0.0702 against 0.0944 of SRA [36].

### 5.1. Ablation Studies

In this section, we present some ablation studies to evaluate the impact of some of the employed design choices. In particular we focus on the addition of noise during training, on the exploitation of the spatial correlation between pixels and finally on the choice of the loss function.

Firstly, we will consider the addition of noise to the simulations. As we have mentioned, there is a loss in performance when switching from synthetic to realistic data, due to the different characteristics of the two sets. The addition of noise helps the generalization capabilities of the network and reduces the gap.

**Figure 9:**
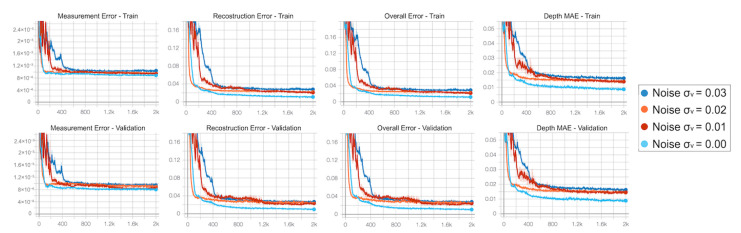
Training curves obtained running the network optimization for noise levels $\sigma_{v}=$ {0.00;0.01;0.02;0.03} on training and validation sets. The metrics monitored are, from left to right, the measurement error, the reconstruction error, the overall error and the MAE on the depth estimated using the predicted output backscattering vector on synthetic data.

**Figure 10:**
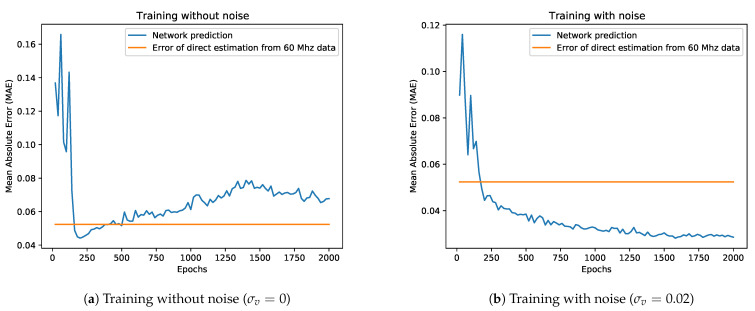
Performance of a network trained on synthetic data with or without noise on the S3 dataset.

We repeated the training multiple times, varying the standard deviation of the noise $\sigma_{v}$ added to the input data. At each epoch we monitored the behaviour of measurement error $L_{m}$, reconstruction error $L_{r}$ and overall error $L=L_{m}+L_{r}$, as well as the MAE on the depth estimated using the predicted output backscattering vector. Figure 9 reports the behaviour of the considered metrics during the optimization on both the synthetic training and validation sets. As expected, the higher the noise level, the larger the errors of the predictive model will be for the synthetic data. More interesting is instead the behaviour on real data: in Figure 10a we show the performance of the network trained without noise on the dataset $S_{3}$ compared to a direct estimation of the depth from the component at 60 MHz. It is clear that, after a promising start, the network performance starts quickly degrading as it better learns the synthetic dataset; as the trend goes on we quickly get to the point where its performance gets worse with respect even to a rough estimation based on a single frequency component. The opposite trend is instead shown in Figure 10b, where we display the network performance over the $S_{3}$ dataset after training the model with a noise std of $\sigma_{v}=0.02$. The network quickly outperforms the naive reconstruction based on a single modulation frequency while showing an overall better behaviour.

In Table 4, we show the performance of our network on the $S_{3}$ dataset for different noise levels. While a small amount of noise helps with the generalization, if it gets too high we instead make the task too hard to solve. Experimentally, a noise std of $\sigma_{v}=0.02$ turned out to be the best compromise, as can be seen in the table.

**Figure 11:**
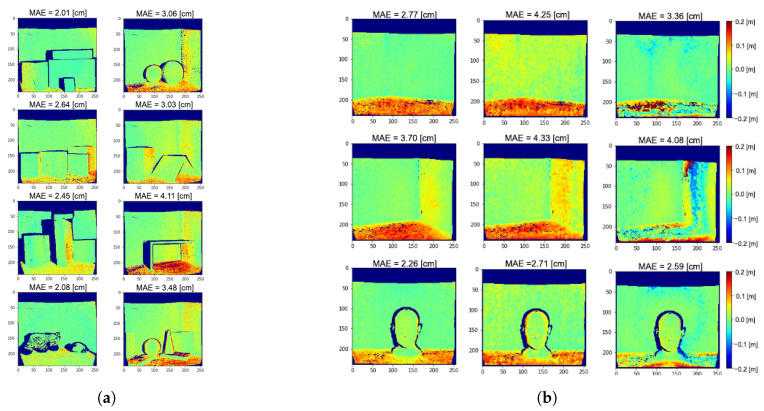
(**a**) Depth error maps on dataset $S_{3}$ obtained without spatial correlation. (**b**) Predicted depth error maps obtained with increasing kernel size on three real scenes, from left to right respectively *P* = 1, 2 and 3.

Another line of investigation is the relevance of the spatial correlation in the final prediction. The idea is that the network should take advantage also from the local information coming from a small neighbourhood around each pixel to produce a more reliable result. To this end, we trained our network for increasing kernel sizes and evaluated its performance on the real datasets. In Table 5 we can see that we have the best results for a window size of 3×3 pixels around the central one, while they get worse for increasing sizes. Focusing on the network trained without spatial correlation (1×1 windows) the average MAEs obtained over $S_{4}$ and $S_{5}$ are respectively 3.43 and 2.52 cm, which turn into 2.99 and 1.88 cm after some bilateral filtering. Experimental results confirm our intuition. In the noise-free case the two networks converge to very similar results, while in the case of noise the spatial correlation helps providing a smoother prediction making the network more resilient against noise. Figure 11a reports the depth error maps we get on dataset $S_{3}$ without exploiting the spatial correlation.

In Figure 11b, we show the results obtained on three real scenes for values of P=1,2 and 3 (corresponding to windows of size 3×3, 5×5 and 7×7). It is quite clear how a bigger window size generates some strong artifacts and leads to over-correcting MPI. One more time we stress the importance of linking the final prediction for each pixel to the corresponding input pixel since it is the one which carries the most information, using only a small amount of spatial correlation to refine the estimation.

To conclude the discussion on spatial information, notice that considering only a very small window size around each pixel also improves the generalization capabilities of the network. As pointed out by the ablation, a direct approach such as the one we attempted (increasing the window size) leads to not very satisfactory performances, but probably the spatial information could be exploited by using a more refined branch (e.g., a second architecture in parallel with a large window size, that only considers the high level structure of the scene, similarly to [24]).

**Figure 12:**
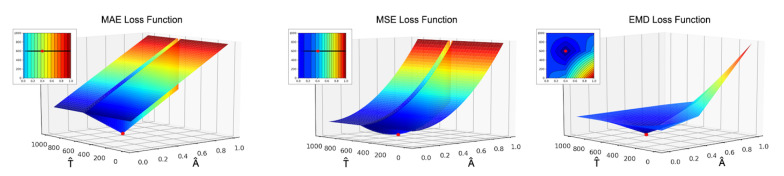
Behaviour of MAE, MSE and Earth Mover Distance (EMD loss functions varying amplitude $\hat{A}$ and position $\hat{T}$ of the predicted direct component).

The choice of a proper loss function is a crucial point in the machine learning pipeline. As already stated, some problems arise with the reconstruction loss function $L_{r}$ since we are dealing with highly sparse vectors and in this case the gradient likely vanishes. In our study we evaluated many different loss function models in order to identify the best suitable one for our task. We started from the common MAE and MSE but they produce an all-zero output backscattering vector in most of the cases. Intuitively, looking at the behaviour of these loss functions in Figure 12 it is evident that applying the gradient descent algorithm the solution easily gets stuck on bad local minima. Then, we shifted to cross-correlation based loss functions obtaining a significant improvement in the final prediction but they still are subjected to numerical instabilities during the optimization phase. The best results came from the weighted Earth Mover Distance introduced in Section 3.2.3 which exhibits good convergence properties and turns out to be able to drive the algorithm towards the optimal solution in a smooth fashion.

## 6. Conclusions

In this paper, we have presented a novel approach for MPI denoising based on transient information. Since neural networks have issues when handling high dimensional data, which is exactly what backscattering vectors are, we split the problem in two parts—a predictive model and a backscattering model. The predictive model takes the input iToF measurements and predicts an encoded version of the transient information, while the backscattering model links each encoded representation to the respective transient one. In this work, the backscattering model has been kept fixed since our aim was MPI denoising rather than transient data reconstruction. In practice, while the direct component has been kept, the global component has instead been summarized into a single peak. This allowed to build a simple but effective model where the neural network only had to predict two peaks from the input raw measurements.

Even if all the trainings have been performed on synthetic data, the testing has been done on real iToF data. Our approach showed close to state of the art performance, without the need of a heavy or complex structure or of a large amount of training data (our model contains only a few thousands parameters).

Our work leaves open several future research directions, first of all an extension of the backscattering model which as of now is quite simple and an extension of the approach employing a similar model for transient data reconstruction. We will also consider more advanced architectures in order to exploit the spatial context in the backscattering estimation.

## Figures and Tables

**Table 1:** Summary of the main state-of-the-art Multi-Path Interference (MPI) correction methods.

	Solution	# of Frequencies	Complexity	MPI Type	Output
Fuchs et al. [33,34]	Iterative	1	High	2-sparse	Depth
Jiménez et al. [35]	Iterative	1	High	2-sparse	Depth
SRA [36]	LP	K>1	Avg	M-sparse	Backscattering
Bhandari et al. [37]	Deterministic	2K+1	High	K-sparse	Depth
Son et al. [18]	FCN		Low	General	Depth + Object Boundary
DeepToF [19]	CNN	1	High	General	Depth
Agresti et al. [24]	CNN	3	Avg	General	Depth
Guo et al. [38]	CNN	3	Avg	General	Depth
Su et al. [17]	CNN	2	High	General	Depth
Agresti et al. [20]	CNN + UDA	3	High	General	Depth
Dong et al. [32]	CNN	1	High	General	Depth
Our Approach	CNN	3	Low	2-sparse	Backscattering

**Table 2:** Properties of the real-world datasets $S_{3},S_{4}$ and $S_{5}$. In this work we only used the data relating to the frequencies marked in bold.

Dataset	Type	Depth GT	Trans. GT	No. Scenes	Spatial Res.	Modulation Frequencies
$S_{3}$	Real	yes	no	8	320×239	10, 20, 30, 40, 50 and 60 MHz
$S_{4}$	Real	yes	no	8	320×239	20, 50 and 60 MHz
$S_{5}$ (*box*)	Real	yes	no	8	320×239	10, 20, 30, 40, 50 and 60 MHz

**Table 3:** Quantitative comparison between several state-of-the-art Multi-Path Interference (MPI) correction algorithms on the real datasets $S_{4}$ and $S_{5}$. Each row reports the depth MAE and the relative error obtained applying the corresponding method w.r.t. the maximum employed frequency (60 MHz for all methods except 20 MHz for [19] (*)).

Method	$S_{4}$ Dataset		$S_{5}$ Dataset	
	MAE	Relative	MAE	Relative
	[cm]	Error	[cm]	Error
Single frequency (20 MHz)	7.28	-	5.06	-
Single frequency (60 MHz)	5.43	-	3.62	-
SRA [36]	5.11	94.1%	3.37	93.1%
DeepToF [19]	5.13	70.5% *	6.68	132% *
+ calibration	5.46	75% *	3.36	66.4% *
Agresti et al. [24]	3.19	58.7%	2.22	60.5%
Agresti et al. [20]	2.36	43.5%	1.66	46.1%
Our Approach	2.79	51.4%	2.27	62.7%
Ours + bilateral filtering	2.60	47.9%	2.12	58.6%
Our Approach (without spatial correlation)	3.43	63.2%	2.52	69.6%
Ours + bilateral filtering	2.99	55.1%	1.88	52.0%

**Table 4:** MAE on the $S_{3}$ dataset for different amounts of noise. Window size is 3×3. The best performance (in bold) is achieved with a noise standard deviation $\sigma_{v}=0.02$.

Mean Absolute Error for Noise with Different Standard Deviations ($\sigma_{v}$)				
$\sigma_{v}$	0.00	0.01	0.02	0.03
MAE [cm]	4.02	2.65	2.58	2.83

**Table 5:** MAE on the $S_{3}$ dataset for different window sizes. Noise level is $\sigma_{v}=0.02$. The best performance (in bold) is achieved with a 3×3 window size.

Mean Absolute Error for Different Window Sizes				
Window size	1×1	3×3	5×5	7×7
MAE [cm]	2.72	2.58	2.61	2.80
